# Impacts of Disruptive Events on Addictive Behavioral Patterns: A Cross‐Sectional Study of Methadone Maintenance Therapy During the COVID‐19 Pandemic in Iran

**DOI:** 10.1002/hsr2.71580

**Published:** 2025-11-30

**Authors:** Zahra Toreyhi, Amir Mohammad Rezai‐Mersagh, Ali Kheradmand, Reyhaneh Mehrvar

**Affiliations:** ^1^ School of Medicine Iran University of Medical Sciences Tehran Iran; ^2^ School of Medicine Shahid Beheshti University of Medical Sciences Tehran Iran; ^3^ Department of Psychiatry, Taleghani Hospital Clinical Research Development Unit, School of Medicine Shahid Beheshti University of Medical Sciences Tehran Iran

**Keywords:** addiction, behavioral patterns, COVID‐19, disruptive events, methadone maintenance treatment

## Abstract

**Background and Aims:**

The COVID‐19 pandemic disrupted healthcare delivery and posed challenges for addiction treatment, particularly methadone maintenance therapy (MMT). This study examined changes in methadone use, behavioral patterns, and psychological well‐being among MMT patients in Tehran, Iran, to assess how public health crises can influence addiction care.

**Methods:**

A retrospective cross‐sectional study with within‐subject pre–post comparisons was conducted among 180 MMT patients from four randomly selected treatment centers. Methadone intake and behavioral outcomes (smoking, physical activity, technology use, and anxiety) were assessed for the periods immediately before and during the pandemic. Multivariable logistic regression identified predictors of dose escalation.

**Results:**

Paired analyses (*n* = 179) showed a modest overall increase in mean methadone dose from 54.16 ± 37.28 mg to 62.09 ± 49.77 mg (mean change +7.93 mg; 95% CI, 2.49–13.37; *p* = 0.004). Subgroup analyses revealed heterogeneity: patients who increased intake (*n* = 57) rose by 35.79 mg (95% CI, 17.38–54.20; *p* < 0.001), while those who decreased intake (*n* = 14) reduced by 37.15 mg (95% CI, –65.73 to –8.57; *p* = 0.005). Regression analysis identified living with family (aOR = 2.72, *p* = 0.01) as a risk factor, while pre‐existing illness (aOR = 0.09, *p* < 0.001) and age 40–50 years (aOR = 0.17, *p* = 0.02) were protective. Methadone formulation, timing, and frequency remained stable. Behaviorally, 21.1% reported stronger drug cravings, 14.4% reported slippage to other substances (most often methamphetamine), 94.4% were smokers with high nicotine dependence, 10% reported reduced physical activity, 44.4% increased mobile phone use, 39.4% increased internet use, and 28.3% experienced high anxiety.

**Conclusion:**

The pandemic was associated with increased methadone use and maladaptive behavioral changes among MMT patients, while dispensing practices remained resilient. Strengthening telehealth capacity, flexible dosing, integrated mental health services, and family‐sensitive counseling will be essential to protect continuity of care during future disruptive events.

## 1 Introduction

Disruptive events—such as pandemics, natural disasters, or conflicts—can destabilize healthcare systems and disproportionately affect vulnerable populations, including those undergoing addiction treatment [1, 2]. Events like the COVID‐19 pandemic posed significant challenges to addiction programs dependent on consistent care, such as methadone maintenance treatment (MMT). Quarantine regulations and infection fears disrupted daily routines, increased psychological stress, and limited access to treatment facilities [3, 4, 5, 6]. With more than 26 million people worldwide living with opioid use disorder (OUD)—a population already at high risk of overdose [7]—the continuity of MMT, a life‐saving intervention that reduces withdrawal, cravings, and relapse, became especially vulnerable during this period [8].

Lazarus and Folkman's Stress and Coping Theory provides the theoretical foundation for this study, describing how individuals respond to crises depending on their perceived control and coping resources [9]. The model has been widely applied in public health research to interpret behavioral adaptations during disruptive events [10] and is particularly relevant to MMT patients facing pandemic‐related stress, disruption, and isolation [11]. In line with global trends, these stressors have been associated with observable behavioral shifts—including increases in smoking, internet use, and substance consumption—reflecting broader links between psychological distress and elevated substance use during crises [12, 13, 14].

In Iran, opioid use disorder constitutes a major public health burden. A large‐scale cohort study reported that 58% of long‐term opiate users met criteria for opioid use disorder according to the Diagnostic and Statistical Manual of Mental Disorders, Fifth Edition (DSM‐5) [15, 16]. National epidemiological data further indicate a substantial unmet treatment need, with approximately half of individuals with drug use disorders and 40% with opioid dependence lacking access to appropriate services [17]. Iran has one of the highest rates of nonmedical opium use globally, and Tehran remains a central hub for treatment demand [18, 19]. During the COVID‐19 pandemic, addiction treatment facilities in the capital experienced reduced in‐person services, regulatory constraints, and limited infrastructure for remote care [20].

While international studies have documented increases in substance use and psychological distress during the pandemic [21], evidence from low‐ and middle‐income countries, particularly in the Middle East, remains scarce [22, 23, 24]. In Tehran, pandemic‐related stress contributed to higher smoking rates, greater reliance on the internet and mobile devices [25, 26], and reduced physical activity, especially among previously active individuals [27]. Although telehealth services were introduced to mitigate access barriers, the overall system response was insufficient, which indicates the need for more resilient and crisis‐adaptive models of addiction care [28].

To address this regional gap, the present study investigates the effects of the COVID‐19 pandemic on methadone use, behavioral patterns, and lifestyle changes among MMT patients in Tehran. It also explores factors influencing these shifts and proposes recommendations for strengthening treatment delivery during disruptive events. In doing so, the study contributes to the achievement of the United Nations Sustainable Development Goal 3 (SDG 3: Ensure healthy lives and promote well‐being for all at all ages), with particular relevance to Target 3.4 (promote mental health and well‐being), Target 3.5 (strengthen prevention and treatment of substance use disorders), and Target 3.8 (achieve universal health coverage including essential services) [29, 30].

## 2 Methods

### 2.1 Study Design

This retrospective cross‐sectional study with within‐subject pre–post comparisons was conducted in Tehran, Iran, in 2021 to examine methadone consumption patterns and related behavioral changes during the COVID‐19 pandemic. Although data were collected at a single timepoint, pre‐pandemic values were obtained retrospectively through self‐report and clinic records, enabling within‐subject comparisons. The design efficiently evaluates multiple variables within a defined timeframe while comparing each participant's pre‐pandemic versus during‐pandemic dose/behaviors. “Pre‐pandemic” was operationalized as the typical dose immediately before the first national COVID‐19 restrictions in Iran; “During‐pandemic” referred to the typical dose/behavior at the time of survey administration after lockdowns. When available, clinic records were used to verify both timepoints and were cross‐checked against self‐report. The approach is appropriate for assessing the consequences of the COVID‐19 public health crisis by providing a snapshot of changes among patients enrolled in MMT programs using quantitative measures.

### 2.2 Research Objectives and Hypotheses

#### 2.2.1 Primary Objective

To identify demographic, clinical, and behavioral factors associated with increased methadone use during the pandemic (vs. no change or decrease) using multivariable regression.

#### 2.2.2 Secondary Objectives

(i) To compare mean methadone dose before and during the pandemic; (ii) To examine shifts in dosage‐range categories (< 40, 40–80, > 80 mg); (iii) To describe concurrent behavioral and lifestyle changes (smoking, physical activity, mobile or internet use, and anxiety).

#### 2.2.3 Hypotheses

Demographic and clinical variables (age group, sex, marital status, living arrangement, history of illness, and smoking status) are associated with increased methadone use (vs. no change or decrease).

Mean methadone dose differs between pre‐pandemic and during‐pandemic periods.

The distribution of patients across dosage‐range categories differs between pre‐pandemic and during‐pandemic periods.

### 2.3 Study Population and Sampling

We used two‐stage cluster sampling, with computer‐generated simple random sampling of eligible participants within each selected center: Tehran was divided into four geographical regions (north, south, east, and west); one addiction treatment center per region was randomly selected. Within each center, eligible participants were randomly drawn from the active registry (unique registry IDs randomized with Excel RAND(); invitations issued in randomized order; non‐responders were replaced using the next randomized IDs). Recruitment continued until the pre‐specified quota for each center was met. No stratification by sex‐ or age was applied at the center level; these variables were handled analytically.

Participants were eligible if they were aged 18 years or older, had been enrolled in an MMT program for ≥ 6 months, and provided informed consent. Individuals were excluded if they had severe psychiatric conditions that could compromise the accuracy of self‐reported data or if they were unwilling to participate.

### 2.4 Measures and Instruments

Data were collected using a structured investigator‐designed questionnaire developed from prior literature to capture context‐specific variables for rapid behavioral assessment under pandemic field constraints [31, 32]. Clinic records were abstracted when available to corroborate methadone doses at both timepoints. The questionnaire included:

#### 2.4.1 Demographics

Age, sex, education, employment, marital status, living arrangement, self‐reported history of illness.

#### 2.4.2 Methadone Use

Dose (mg/day), formulation (syrup/tablet), timing, dosing frequency recorded for both periods; clinic records were used for verification where available.

Participants were classified as “increase,” “decrease,” or “unchanged” based on the direction of the paired pre–post dose difference; where clinic records and self‐report disagreed, clinic records were prioritized.

#### 2.4.3 Smoking Behavior

“time to first cigarette after waking” from the Fagerström Test for Nicotine Dependence (FTND), used as a validated single‐item indicator of nicotine dependence [33, 34].

#### 2.4.4 Physical Activity

Single investigator‐developed item on perceived change due to COVID‐19 (increase/decrease/no change) to minimize burden and recall bias during clinic visits [35].

#### 2.4.5 Technology Use (Mobile/Internet)

Single‐item questions on (i) increased use, (ii) disruption of daily functioning, and (iii) discomfort when unable to access, chosen for feasibility in pandemic settings [36, 37].

#### 2.4.6 Anxiety

Single self‐report item on high anxiety during the pandemic, a pragmatic indicator consistent with large‐scale COVID‐19 behavioral surveys [38].

Single‐item measures such as these have been widely adopted in behavioral and addiction research to enable rapid yet valid assessment. For example, a single‐item measure of loneliness demonstrated moderate convergent validity with a validated multi‐item scale, and a single‐item readiness‐to‐change ruler showed predictive validity comparable to longer readiness measures. Importantly, in substance‐use disorder treatment, single‐item motivational measures predicted treatment outcomes with validity comparable to multi‐item tools [39]. Methodological evidence further indicates that single‐item ratings of mood and health can provide results consistent with traditional multi‐item questionnaires, supporting their use in large‐scale or time‐constrained surveys [40]. Similarly, during the COVID‐19 pandemic, single‐item measures of perceived vulnerability were effectively used to capture risk perception in rapid behavioral surveys [31].

### 2.5 Statistical Analysis

All analyses were conducted using *IBM SPSS Statistics for Windows, Version 22.0* (IBM Corp., Armonk, NY, USA). Analyses were based on available data; paired comparisons were performed on complete pre–post pairs, with the reported sample size (n) for each analysis. Because only one paired methadone dose was missing, no imputation was performed.

#### 2.5.1 Primary Analysis

Multivariable logistic regression was used to identify factors associated with increased methadone use (vs. no change/decrease) during the pandemic. Results are presented as adjusted odds ratios (aORs) with 95% confidence intervals (CIs). The following covariates were included in the model: age group, sex, marital status, living arrangement, history of illness, and smoking status.

#### 2.5.2 Secondary Analyses

(i) Paired *t*‐tests compared mean methadone dose pre‐ versus during‐pandemic (within‐person differences), with mean difference and 95% CI (ii) *χ*² tests (with Fisher's exact when expected counts < 5) assessed categorical distributions across periods (e.g., dosage ranges, formulation, timing), with effect sizes reported as Cramér's V.

Because most behavioral and psychological measures were categorical or ordinal, correlations were examined using *χ*² with Cramér's *V* rather than Pearson's *r*, and selected variables were included in the regression model based on conceptual relevance and statistical criteria.

#### 2.5.3 Reporting Conventions

All tests were two‐sided (*α* = 0.05). *p*‐Values were reported following journal guidelines: *p* < 0.001 when < 0.001; 0.001–0.01 to three decimals; ≥ 0.01 to two decimals; > 0.99 as *p* > 0.99. Effect sizes and CIs are presented alongside *p*‐values to convey both precision and magnitude. Figures display 95% CIs, and tables report SDs where applicable. Statistical reporting follows SAMPL guidance, and observational reporting adheres to STROBE guidelines.

#### 2.5.4 Ethics

This study was approved by the Ethics Committee of Shahid Beheshti University of Medical Sciences (Approval Code: IR.SBMU.MSP.REC.1400.413). All participants provided written informed consent before participation. Confidentiality and anonymity were assured, and participants were informed of their right to withdraw from the study at any time without any impact on their treatment. Given the ongoing COVID‐19 pandemic during the time of data collection, ethical challenges related to participant safety and voluntary engagement were addressed by implementing health protocols and clearly communicating the voluntary nature of participation in a safe and respectful manner.

## 3 Results

A total of 180 individuals undergoing MMT participated in the study; paired dose analyses used *n* = 179 due to one missing pre/post value. Baseline demographic and clinical characteristics are summarized in Table 1. The majority were male (95.0%), with 61.7% married or divorced, and 56.1% had completed only high school education. One‐third (31.7%) were aged 40–50 years, and 32.8% were over 50 years.

**Table 1:** Basic demographic and clinical characteristics of patients undergoing methadone maintenance treatment in selected addiction treatment centers in Tehran (*n* = 180). Values are presented as numbers (percentages).

Variable	Category	Frequency (%*n*)
Age	Under 30 years	16 (8.9)
	30–40 years	48 (26.7)
	40–50 years	57 (31.7)
	Over 50 years	59 (32.8)
Sex	Male	171 (95.0)
	Female	9 (5.0)
Education	High school	101 (56.1)
	Diploma and postgraduate diploma	61 (33.9)
	Bachelor's degree and higher	18 (10)
Occupation	Employee	34 (18.9)
	Freelance job	95 (53.3)
	Unemployed and housewife	50 (27.8)
Marital status	Single	69 (38.3)
	Married/divorced	111 (61.7)
Living arrangement	With family	108 (60.0)
	Not with family	72 (40.0)
History of Illness	Yes	18 (10.0)
	No	162 (90.0)

### 3.1 Primary Analysis

#### 3.1.1 Multivariable Logistic Regression

**Figure 1:**
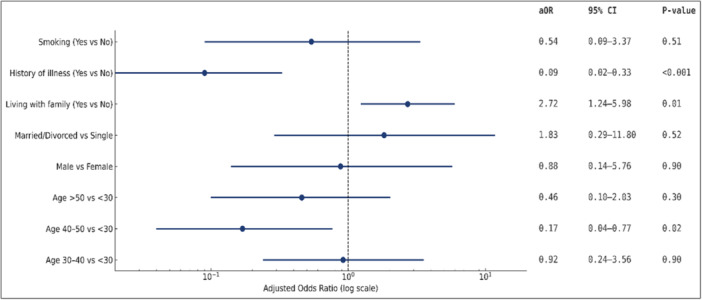
Multivariable logistic regression of factors associated with increased methadone use during the COVID‐19 pandemic (*n* = 179). Adjusted odds ratios (aOR) with 95% confidence intervals (CIs) are shown on a logarithmic scale; the dashed vertical line indicates the null value (aOR = 1). *p*‐values are provided for each predictor. Reference groups: < 30 years (age), female (sex), single (marital status), nonsmoker (smoking), no history of illness, and not living with family. Significant associations (*p* < 0.05) are observed for history of illness, living with family, and age 40–50 years.

Results are presented in Table 2 and illustrated in Figure 1. Three factors were independently associated with increased methadone use during the pandemic. Compared with patients under 30 years, those aged 40–50 years had significantly lower odds of increasing their dose (aOR = 0.17; 95% CI, 0.04–0.77; *p *= 0.02). Living with family was positively associated with dose increase (aOR = 2.72; 95% CI, 1.24–5.98; *p *= 0.01), while a history of illness was strongly protective (aOR = 0.09; 95% CI, 0.02–0.33; *p *< 0.001). Other covariates, including sex, marital status, and smoking, were not statistically significant. Overall, pairwise associations among demographic and clinical variables were weak to moderate (Cramér's *V* < 0.30), indicating low multicollinearity. These findings together with the regression results, show three significant predictors of dose escalation with living with family and history of illness as strong predictors, and age 40–50 years as strong protective factor compared with those under 30 years.

**Table 2:** Multivariable logistic regression of factors associated with increased methadone use during the COVID‐19 pandemic (*n* = 179).

Variable	aOR (95% CI)	*p* value
Age (Ref: < 30 years)		
30–40 years	0.92 (0.24–3.56)	0.90
40–50 years	**0.17 (0.04–0.77)**	0.02
> 50 years	0.46 (0.10–2.03)	0.30
Sex (male vs. female)	0.88 (0.14–5.76)	0.90
Marital status (married/divorced vs. single)	1.83 (0.29–11.80)	0.52
Living with family (yes vs. no)	**2.72 (1.24–5.98)**	0.01
History of illness (yes vs. no)	**0.09 (0.02–0.33)**	< 0.001
Smoking (yes vs. no)	0.54 (0.09–3.37)	0.51

*Note:* Bold values indicate statistical significance at *α* = 0.05.

Abbreviations: aOR, adjusted odds ratio; CI, confidence interval; Ref, reference category.

### 3.2 Secondary Analyses

#### 3.2.1 Methadone Dose Comparisons

**Figure 2:**
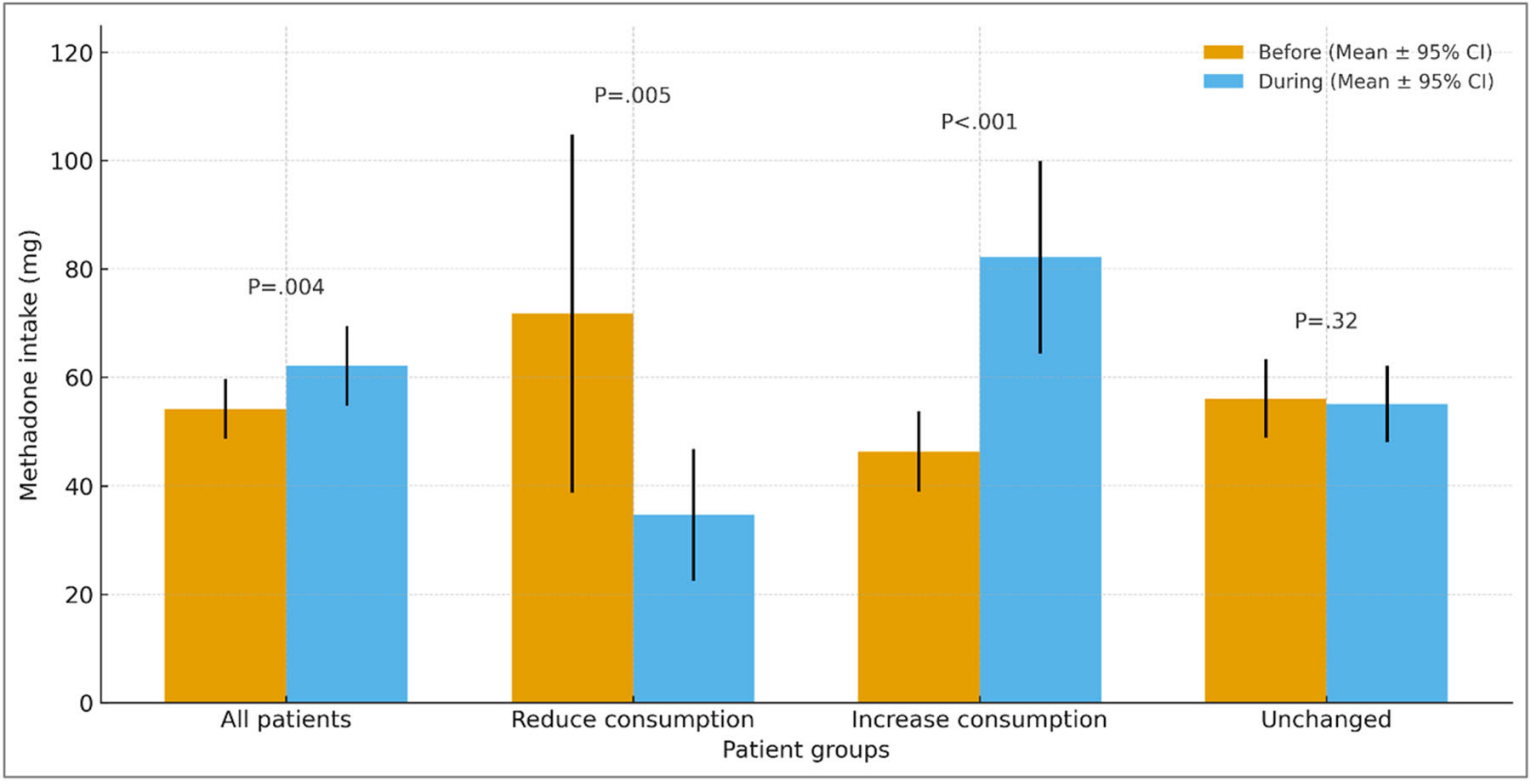
Comparison of methadone intake before and during the COVID‐19 pandemic among patients receiving methadone maintenance treatment. Bars represent mean daily dose; error bars show 95% confidence intervals (CIs). Two‐sided *p*‐values from paired *t*‐tests (*α* = 0.05) are annotated above each group. Paired analyses used complete pre–post pairs (*n* = 179; one missing pair); subgroup ‐ sizes: Reduce (*n* = 14), Increase (*n* = 57), Unchanged (*n* = 108). (Yellow bar = before; Blue bar = during).

Across the 179 patients with paired data, mean methadone dose increased from 54.16 ± 37.28 mg prepandemic to 62.09 ± 49.77 mg during the pandemic, a mean rise of 7.93 mg (95% CI, 2.49–13.37; *p *= 0.004; Table 3, Figure 2). Subgroup analyses showed heterogeneity. Participants reporting decreased intake (*n* = 14) had a mean reduction of 37.15 mg (95% CI, –65.73 to –8.57; *p *= 0.005). In contrast, those reporting increase (*n* = 57) rose by 35.79 mg (95% CI: 17.38–54.20; *p *< 0.001). The unchanged group (*n* = 108) showed no meaningful difference (*p *= 0.32).

**Table 3:** Comparison of methadone intake before and during the COVID‐19 pandemic among patients undergoing methadone maintenance treatment in Tehran, Iran (*n* = 179).

Variable	Number	Before COVID‐19, mean (SD)	During COVID‐19, mean (SD)	Mean difference (95%CI)	*p* value
All patients	179	54.16 (37.28)	62.09 (49.77)	+7.93 (+2.49 to +13.37)	0.004
Reduced consumption	14	71.79 (57.20)	34.64 (21.07)	–37.15 (–65.73 to –8.57)	0.005
Increased consumption	57	46.32 (28.01)	82.11 (67.16)	+35.79 (+17.38 to +54.20)	< 0.001
Unchanged consumption	108	56.02 (37.78)	55.09 (36.66)	–0.93 (–2.78 to +0.92)	0.32

*Note:* Values are presented as mean ± SD. Mean differences represent post–pre changes, with positive values indicating increases and negative values indicating decreases. Paired comparisons used complete pre–post pairs (overall *n* = 179; Reduced *n* = 14; Increased *n* = 57; Unchanged *n* = 108). Two‐sided *p*‐values from paired *t*‐tests (*α* = 0.05) were used.

#### 3.2.2 Dosage Range Distribution

**Figure 3:**
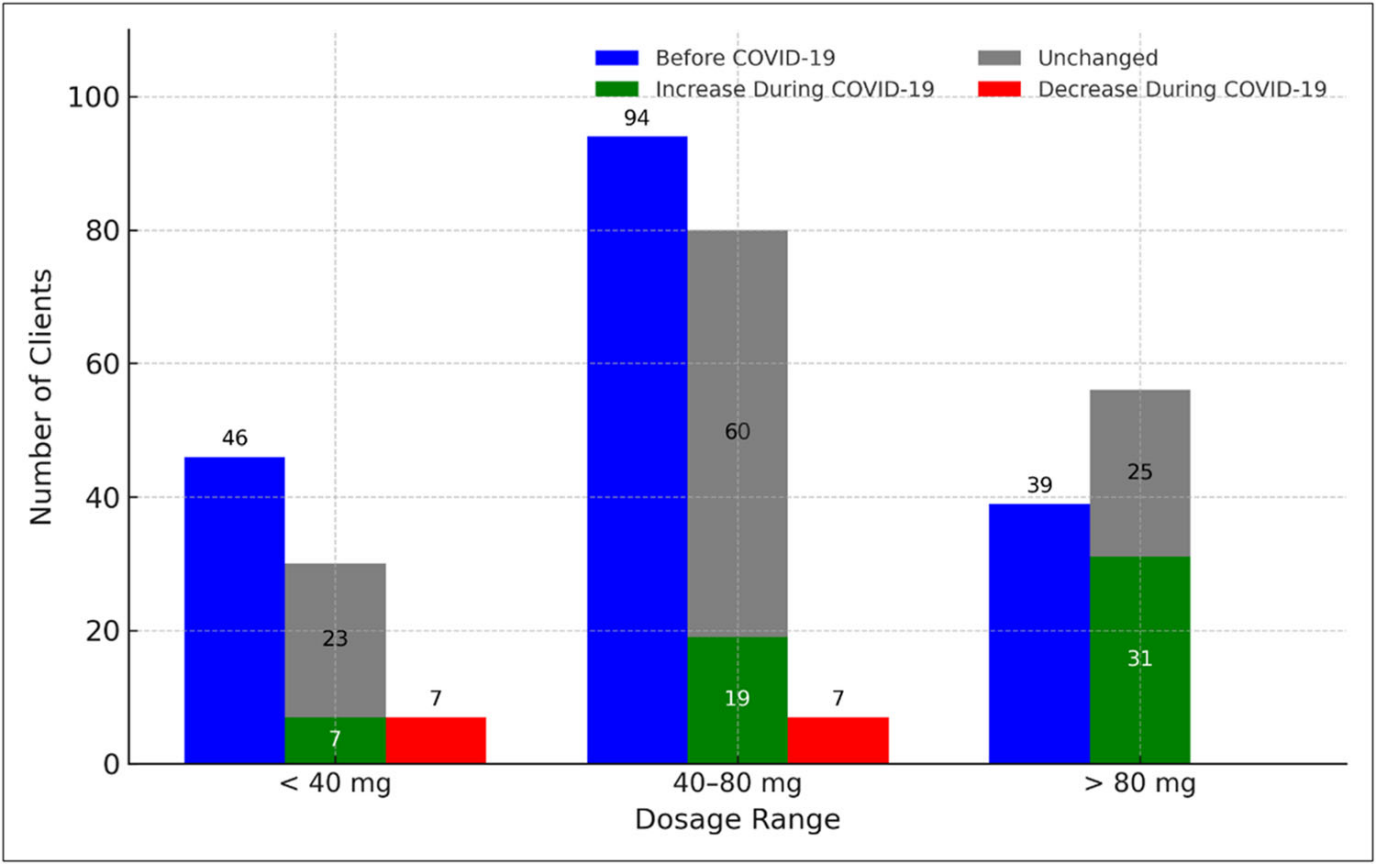
Distribution of methadone consumption across dosage ranges (< 40 mg, 40–80 mg, > 80 mg) before and during the COVID‐19 pandemic. Blue bars represent the baseline distribution before the pandemic, while green (increased), red (decreased), and gray (unchanged) bars indicate participants' consumption categories during the pandemic. Values reflect the number of patients within each subgroup. This visualization highlights dosage shifts among methadone maintenance treatment (MMT) patients. mg, milligrams.

Table 4 and Figure 3 display methadone use categorized into dosage ranges. Before the pandemic, distributions did not differ significantly across the three groups (*χ*²(4) = 0.16, *p* > 0.99; Cramér's *V* ≈ 0.02, negligible effect). During the pandemic, significant shifts were observed in distributions (*χ*²(4) = 26.1, *p *< 0.001; Cramér's *V* = 0.281, medium effect). Most patients in the increase group shifted into the > 80 mg category, while none in the reduction group remained in that range.

**Table 4:** Changes in methadone dosage levels before and during the COVID‐19 pandemic among patients undergoing methadone maintenance treatment in Tehran (*n* = 179).

Dosage range (mg)	Reduced *n* (%)	Increased *n* (%)	Unchanged *n* (%)	*χ*² (*df*)	*p* value	Cramér's *V*
Before COVID‐19				*χ*²(4) = 0.16	> 0.99	0.02
< 40 mg	2 (14.3)	21 (36.8)	23 (21.1)	—	—	—
40–80 mg	8 (57.1)	26 (45.6)	60 (55)	—	—	—
> 80 mg	4 (28.6)	10 (17.5)	25 (23.9)	—	—	—
During COVID‐19				*χ*²(4) = 26.1	< 0.001	0.281
< 40 mg	7 (50.0)	7 (12.3)	23 (21.1)	—	—	—
40–80 mg	7 (50.0)	19 (33.3)	60 (55.6)	—	—	—
> 80 mg	0 (0.0)	31 (54.4)	25 (22.9)	—	—	—

*Note:* Values are presented as number (percentage). Comparisons across intake groups were performed using chi‐square tests; effect sizes reported as Cramér's *V*. (before COVID: 0.02, negligible; during COVID: 0.281, medium).

#### 3.2.3 Usage Patterns

**Figure 4:**
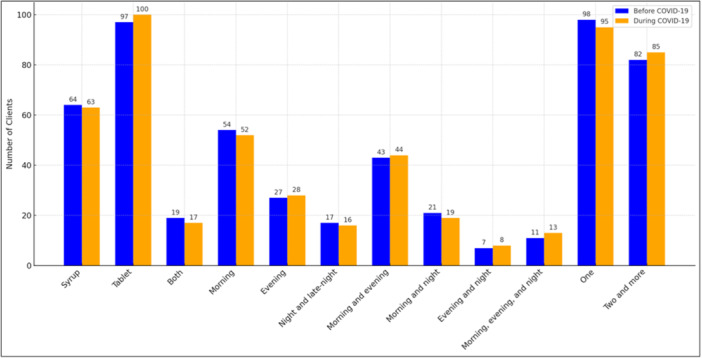
Comparison of methadone administration modalities, timing, and frequency before and during the COVID‐19 pandemic. Bars represent the number of patients using each form of methadone (syrup, tablet, or both), intake timing (e.g., morning, evening, combined schedules), and dosing frequency (once vs. twice or more daily), before (blue) and during (orange) the pandemic. Statistical differences were assessed using Chi‐square tests: drug form (*χ*²(2) = 0.16, *p* = 0.92, Cramér's *V* = 0.021), timing (*χ*²(6) = 1.23, *p* = 0.98, *V* = 0.058), and dosing frequency (*χ*²(1) = 0.003, *p* = 0.96, *V* = 0.004) were not statistically significant. COVID‐19, coronavirus disease 2019.

Methadone formulation, intake timing, and dosing frequency remained stable (Table 5 and Figure 4). Syrup versus tablet use showed no change (*χ*²(2) = 0.16; *p *= 0.92; Cramér's *V* = 0.021). Timing of intake across seven categories was unchanged (*χ*²(6) = 1.23; *p* = 0.98; Cramér's *V* = 0.058). Dosing frequency was similar pre‐ and during‐pandemic (*χ*²(1) = 0.003; *p* = 0.96; Cramér's *V* = 0.004).

**Table 5:** Comparison of methadone usage patterns before and during the COVID‐19 pandemic among patients in Tehran, Iran (*n* = 180).

Variable	Before COVID‐19 *n* (%)	During COVID‐19 *n* (%)	*χ*² (*df*)	*p* value	Cramér's *V*
Form			*χ*²(2) = 0.16	0.92	0.021
Syrup	64 (35.6)	63 (35.0)			
Tablet	97 (53.9)	100 (55.6)			
Both	19 (10.6)	17 (9.4)			
Timing of intake			*χ*²(6) = 1.23	0.98	0.058
Morning	54 (30.0)	52 (28.9)			
Evening	27 (15.0)	28 (15.6)			
Night and late‐night	17 (9.4)	16 (8.9)			
Morning and evening	43 (23.9)	44 (24.5)			
Morning and night	21 (11.7)	19 (10.6)			
Evening and night	7 (4.0)	8 (4.5)			
Morning, evening, and night	11 (6.2)	13 (7.4)			
Doses per day			*χ*²(1) = 0.003	0.96	0.004
One	98 (54.4)	95 (52.8)			
Two and more	82 (45.6)	85 (47.2)			

*Note:* Values are presented as number (percentage). *p*‐values in section headings represent overall comparisons between the two time periods (before and during the pandemic), calculated using the Chi‐square test. Sub‐rows show the distribution within each category.

#### 3.2.4 Behavioral and Lifestyle Changes

**Figure 5:**
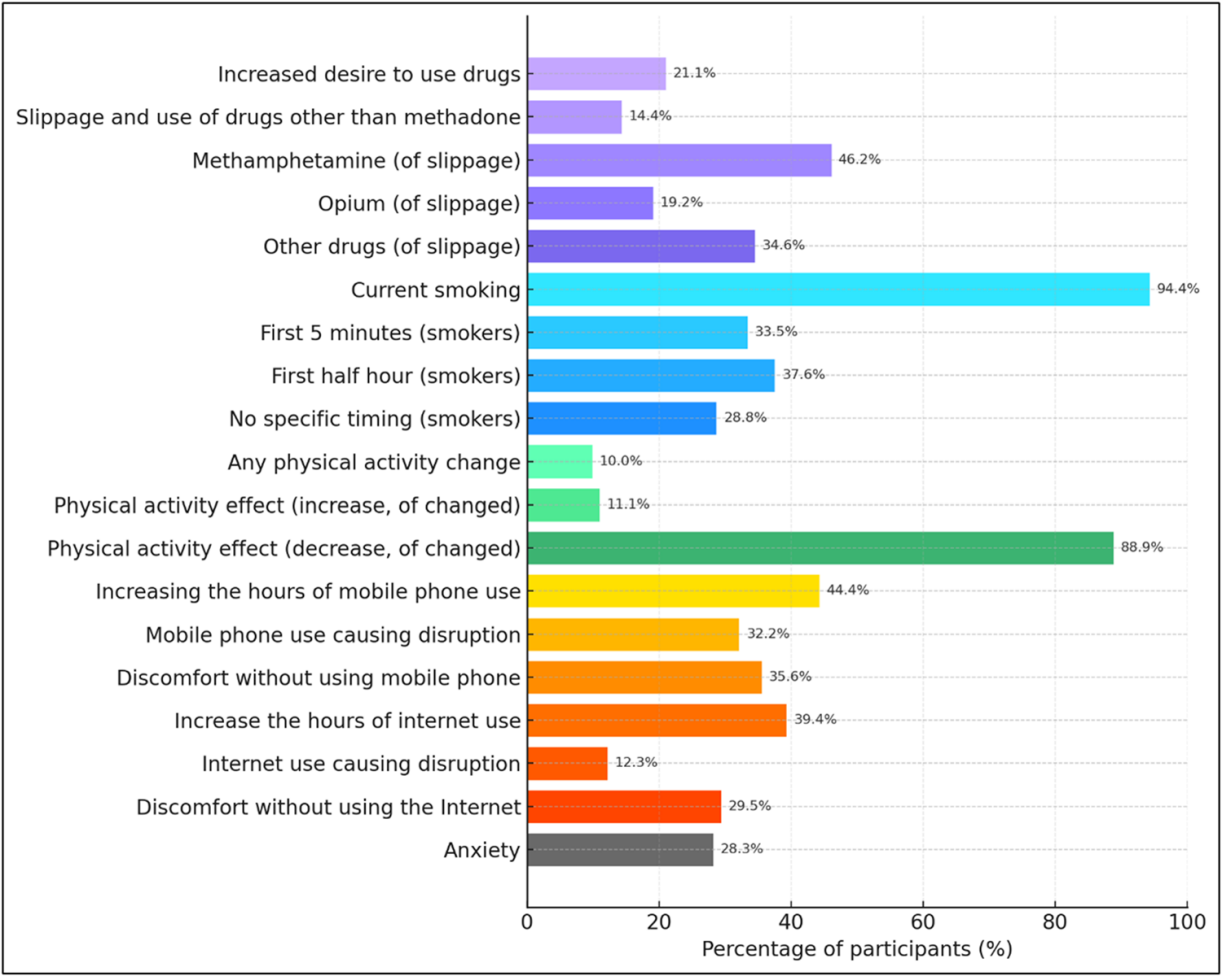
Behavioral and lifestyle changes among methadone maintenance treatment (MMT) participants during the COVID‐19 pandemic. Bars represent the percentage of participants reporting changes in substance use, smoking habits, physical activity, mobile phone and internet use, and anxiety. Subgroup percentages are shown for participants who reported slippage (*n* = 26), current smokers (*n* = 170), and those with a change in physical activity (*n* = 18). COVID‐19, coronavirus disease 2019; MMT, methadone maintenance treatment.

Table 6 and Figure 5 summarize behavioral changes during the pandemic. A total of 21.1% of participants reported a stronger desire to use drugs, and 14.4% reported slippage to substances other than methadone, most often methamphetamine (46.2%). Smoking remained highly prevalent (94.4%), with one‐third reporting smoking within 5 min of waking. Physical activity decreased in 10% overall, with 16 of 18 reporting a reduction. Technology‐related behaviors also changed; 44.4% of participants reported increased mobile phone use, 32.2% reported disruption of daily functioning, and 35.6% reported discomfort without phone access. Internet use increased in 39.4% of participants, while 12.3% reported that it disrupted daily life and 29.5% experienced discomfort when unable to access it. Finally, 28.3% of participants reported high anxiety levels.

**Table 6:** Behavioral and lifestyle changes during the COVID‐19 pandemic among patients receiving methadone maintenance treatment in Tehran, Iran (*n* = 180).

Variable	*n* (%)
Increased desire to use drugs	38 (21.1)
Slippage and use of drugs other than methadone	26 (14.4)
Other types of materials	
Methamphetamine	12 (46.2)
Opium	5 (19.2)
Other drugs	9 (34.6)
Current Smoking	170 (94.4)
Time to first cigarette	
Within 5 min	57 (33.5)
Within 30 min	64 (37.6)
No specific timing	49 (28.8)
Any physical activity change	18 (10.0)
Increased	2 (11.1)
Decreased	16 (88.9)
Technology‐related behaviors	
Increased mobile phone use	80 (44.4)
Mobile phone use causing disruption	58 (32.2)
Discomfort without mobile phone	64 (35.6)
Increased internet use	71 (39.4)
Internet use causing disruption	22 (12.3)
Discomfort without internet	53 (29.5)
High anxiety	51 (28.3)

*Note:* Values are number (percentage). Variables reflect participant self‐reports regarding behavioral and lifestyle changes during the pandemic.

Time‐to‐first‐cigarette rows are among current smokers (*n* = 170); “Increased/Decreased physical activity” percentages are within those reporting any change (*n* = 18); “of slippage” percentages are within participants who reported slippage (*n* = 26).

## 4 Discussion

This study examined the impact of COVID‐19 pandemic on methadone dosing and related behavioral patterns among patients enrolled in MMT programs in Tehran. Using paired pre–post comparisons combined with multivariable regression, we observed a modest overall increase in methadone dose, with clinically meaningful upward shifts in a subset of patients who escalated to high‐dose categories. In contrast, formulation, timing and dosing frequency remained stable. Regression analysis identified specific predictors of dose escalation, including living with family (higher odds), history of illness (protective), and younger age (< 40 years). Behavioral outcomes revealed elevated drug craving, slippage into non‐methadone substances, pervasive smoking, decreased physical activity, increased technology dependence, and heightened anxiety within the pandemic. Together, these findings highlight both the resilience of core dispensing practices and the vulnerability of patients to dose escalation and maladaptive coping during a public health crisis.

### 4.1 Primary Findings and Interpretation

Regression results showed that living with family increased the likelihood of methadone dose escalation, while pre‐existing illness and being in the 40–50 age group were protective. The forest plot also suggests a gradient of decreasing odds of dose escalation with increasing age, although statistical significance was only observed in the 40–50 year group. These patterns emphasize the dual role of social context and health status in shaping dose trajectories. Family presence can provide support but may also impose stressors that heighten withdrawal risk or encourage higher dosing as a stabilizing strategy, consistent with prior evidence that family dynamics can either reinforce or mitigate substance use [41, 42, 43]. In high‐stress contexts, substance use often serves as a coping mechanism for stress relief [44]. Conversely, patients with pre‐existing illnesses may have been more cautious or under closer medical supervision, limiting dose escalation. The protective effect observed in the 40–50 age group may reflect greater psychosocial stability compared with younger patients, who tend to rely more on emotion‐focused coping strategies associated with increased substance use [45, 46]. Viewed through the lens of *Stress and Coping Theory*, these results suggest that coping resources and perceived stress shaped methadone use trajectories during the pandemic. At the same time, Figure 5 shows that several nonsignificant predictors, including sex and marital status, had wide confidence intervals, indicating limited power for these covariates and warranting cautious interpretation of their null results.

### 4.2 Methadone Dose Escalation

Although the mean increase in methadone dose was modest (+7.93 mg), subgroup analysis revealed pronounced heterogeneity. Patients in the “increase” subgroup showed substantial rises (+35.8 mg), with more than half shifting into the > 80 mg category—a level associated with higher tolerance and potential safety concerns. These changes likely reflect pandemic‐induced stress, uncertainty, and disruption of daily routines. By contrast, the small subgroup who reduced their methadone dose (*n* = 14) began the pandemic with relatively higher baseline intake and showed large downward adjustments. This pattern is consistent with dose retitration after missed visits or clinical concerns about safety at higher levels, both of which have been reported during service disruptions. Another possibility is that some patients experienced fewer external triggers for opioid craving during lockdown, reducing their need for higher doses. Importantly, none of these patients remained in the > 80 mg category, reinforcing the interpretation that downward adjustments were primarily related to safety and treatment regulation rather than disengagement. Together, these findings illustrate that while most patients required higher doses to remain stable, a smaller subgroup underwent dose reductions for safety or contextual reasons. Similar trends were reported internationally, where take‐home allowances expanded and telehealth became more widely used, but clinicians simultaneously increased dosing to prevent relapse and withdrawal [47, 48, 49]. These parallels underscore the need to balance service flexibility with rigorous dose safety monitoring during future crises.

### 4.3 Stability of Dispensing Patterns

Despite these dose changes, methadone formulation, timing, and frequency of administration remained unchanged across the study period. This stability suggests that Tehran's treatment centers preserved essential dispensing practices despite pandemic constraints, buffering against severe access disruptions. The absence of shifts in formulation (syrup vs. tablet), intake timing, or daily dosing frequency highlights operational resilience, suggesting that centers prioritized stability in daily treatment routines even as broader societal functions were destabilized. Such structural continuity is particularly significant in settings with limited telehealth infrastructure, where disruptions in in‐person dispensing could have had severe consequences for retention in care, and it has been recognized as critical for maintaining treatment adherence and preventing destabilization during emergencies [50, 51, 52, 53, 54, 55, 56, 57]. While our study did not directly measure relapse or diversion, the preservation of dispensing practices likely served as a protective factor and cornerstone of treatment resilience by reducing disengagement risk at a time of heightened stress and vulnerability—not only during pandemics but also in other disruptive events such as natural disasters or political instability.

### 4.4 Behavioral and Lifestyle Adaptations

Family context is a recognized determinant of addiction trajectories. As Klagsbrun et al. noted, family dynamics can both reinforce use and scaffold recovery [41]. In our study, living with family was independently associated with higher odds of methadone dose escalation, consistent with the idea that close‐quarters support during a crisis can simultaneously introduce stressors that intensify craving or destabilize dosing routines [41].

The pandemic's psychological toll was reflected in multiple behavioral shifts. Craving substances increased in 21.1% of participants, and 14.4% reported slippage to non‐methadone substances, most commonly methamphetamine (46.2% among those reporting slippage). Smoking remained highly prevalent (94.4%), with 71.1% of smokers lighting their first cigarette within 30 min of waking (33.5% within 5 min; 37.6% within 30 min), reflecting high nicotine dependence per the FTND anchor item. Over one‐quarter reported increased consumption, a pattern consistent with Vanderbruggen et al., who observed comparable rises in tobacco use linked to stress and unemployment during COVID‐19 restrictions [58].

Technology‐related changes were also prominent. Nearly half of participants reported increased mobile phone (44.4%) and internet use (39.4%), mirroring global reports of pandemic‐driven digital reliance [59, 60]. In our study, these behaviors were not benign: 32.2% reported phone use disrupting daily functioning, 12.3% reported internet disruption, and 35.6% and 29.5% reported discomfort when access was restricted. These findings illustrate how psychosocial stressors manifested in maladaptive coping even when treatment continuity was preserved, reinforcing the view that crises magnify vulnerabilities through both direct stress exposure and indirect behavioral consequences [61, 62, 63].

The insights gained from this study advance SDG 3 by elucidating strategies to strengthen mental health support, maintain addiction treatment continuity, and promote healthcare resilience during public health crises. Nearly one‐third of participants (28.3%) reported high anxiety, echoing international evidence that pandemic‐related distress stemmed from infection fears, bereavement, financial insecurity, and social isolation [13, 14, 57, 58, 62, 63].

Such stressors likely amplified maladaptive behaviors in our study, including increased smoking, stimulant slippage, and technology reliance, underscoring the need for integrated psychosocial care within addiction treatment. From a clinical perspective, embedding nicotine‐dependence treatment, stimulant‐use screening, and structured mental health support into MMT programs, alongside counseling on technology use and physical activity, could mitigate these risks [61]. At the systems level, flexible protocols such as telemedicine and adjustable dosing schedules, coupled with explicit inclusion of addiction services in emergency preparedness plans, are essential to safeguard continuity of care. These measures align with SDG 3 targets—particularly Target 3.4 (mental health promotion) and Target 3.5 (substance‐use disorder treatment access)—and emphasize the importance of resilient, integrated approaches to addiction care during future disruptive events.

### 4.5 Limitations

This study has several limitations. First, reliance on self‐reported measures may have introduced recall or social desirability bias. Second, the cross‐sectional design precludes causal inference between the pandemic and observed behavioral or dosing changes. Third, unmeasured confounders such as socioeconomic status, psychiatric comorbidities, and prior treatment history may have influenced outcomes. In addition, we did not adjust for potential clustering by treatment center, which may have slightly underestimated variance. Finally, findings are specific to treatment centers in Tehran and may not generalize to other regions or health systems. Despite these constraints, the use of the structured questionnaire, combined with randomized cluster sampling across diverse city zones and incorporation of clinic record verification, where available, enhances the representativeness and provides a robust snapshot of treatment and behavioral patterns during the pandemic.

### 4.6 Further Suggestions

Several practical steps can be drawn from these findings. First, flexible treatment protocols—such as adjustable dosing schedules, expanded telemedicine, and family‐inclusive counseling—can help sustain continuity of care. Second, integration of mental health services into MMT programs is essential to address pandemic‐related stress, anxiety, and coping failures. Third, digital platforms should be leveraged for remote counseling and peer support, while monitoring for problematic overuse. Finally, public health preparedness initiatives should explicitly recognize addiction treatment as an essential service during emergencies, ensuring uninterrupted access to methadone and psychosocial care.

## 5 Conclusions

This study found that the COVID‐19 pandemic was associated with increases in methadone use and maladaptive behavioral changes among MMT patients in Tehran, while dispensing practices remained stable. The overall rise in methadone dose was modest, but a subset of patients escalated into higher‐dose categories, raising potential safety concerns. Younger age and living with family were linked to dose escalation, whereas individuals with pre‐existing illness were less likely to increase their dose. These findings underscore the complex interaction of demographic, clinical, and social factors that shape treatment trajectories during disruptive events. In addition to pharmacological changes, the pandemic was accompanied by behavioral and psychological consequences. Participants reported elevated craving, slippage to non‐methadone substances, high levels of nicotine dependence, reduced physical activity, and increasing reliance on mobile phones and the internet. Nearly one‐third of patients described heightened anxiety, reflecting the psychosocial toll of prolonged uncertainty, isolation, and disruption. These maladaptive patterns point to the broader vulnerability of individuals receiving MMT, even when treatment continuity is preserved.

At the same time, the stability of methadone formulation, timing, and dosing frequency suggests that treatment centers demonstrated resilience by maintaining core dispensing practices despite pandemic restrictions. This continuity likely protected against more severe disruptions in access and underscores the importance of safeguarding operational capacity in addiction services during crises. The dual picture of resilience in service delivery and vulnerability in patient behavior highlights the need for integrated responses that address both structural and psychosocial dimensions of care.

To prepare for future disruptive events, several priorities emerge from these findings. Addiction treatment systems should adopt flexible dosing schedules, strengthen telehealth infrastructure, and integrate mental health support within MMT programs. Family‐sensitive counseling may also help mitigate stressors associated with close‐quarters living. Beyond individual interventions, public health preparedness should explicitly designate addiction care as an essential service to ensure uninterrupted access to methadone and related supports during emergencies. By documenting these patterns in a middle‐income setting where evidence is scarce, this study contributes to the global understanding of how crises affect vulnerable populations receiving addiction treatment. Implementing its recommendations will help enhance the resilience and equity of MMT programs, in line with the objectives of Sustainable Development Goal 3, particularly the promotion of mental health, the prevention and treatment of substance use disorders, and the strengthening of universal access to essential health services.

## Author Contributions

Conceptualization: Zahra Toreyhi and Amir Mohammad Rezai‐Mersagh. Methodology: Zahra Toreyhi and Amir Mohammad Rezai‐Mersagh. Formal analysis: Zahra Toreyhi. Investigation: Zahra Toreyhi and Amir Mohammad Rezai‐Mersagh. Data curation: Zahra Toreyhi. Writing – original draft: Zahra Toreyhi and Reyhaneh Mehrvar. Writing – review and editing: Reyhaneh Mehrvar. Supervision: Ali Kheradmand and Reyhaneh Mehrvar. All authors have read and approved the final manuscript.

## Conflicts of Interest

The authors declare no conflicts of interest.

## Transparency Statement

The lead authors, Ali Kheradmand and Reyhaneh Mehrvar affirm that this manuscript is an honest, accurate, and transparent account of the study being reported; that no important aspects of the study have been omitted; and that any discrepancies from the study as planned (and, if relevant, registered) have been explained.

## Data Availability

The data that support the findings of this study are available within the article. Additional data can be made available from the corresponding authors upon reasonable request and with appropriate institutional approval.
